# Corneal Confocal Microscopy Identifies and Differentiates Patients With Multiple Sclerosis and Epilepsy

**DOI:** 10.1167/tvst.13.12.22

**Published:** 2024-12-13

**Authors:** Ioannis N. Petropoulos, Kareem Essam Aly, Shaikha Al-Thani, Georgios Ponirakis, Hoda Gad, Adnan Khan, Beatriz Canibano, Dirk Deleu, Naveed Akhtar, Gayane Melikyan, Boulenouar Mesraoua, Maria Siddiqi, Jon Perkins, Novsheen Mir, Reny Francis, Abdul Salam, Ahmed El-Sotouhy, Surjith Vattoth, Ahmed Own, Saadat Kamran, Rayaz A. Malik

**Affiliations:** 1 Division of Research, Weill Cornell Medicine Qatar, Doha, Qatar; 2 Emergency Medicine, Hamad Medical Corporation, Doha, Qatar; 3 Faculty of Health Sciences, Khyber Medical University, Peshawar, Pakistan; 4 Department of Neurology, Hamad Medical Corporation, Doha, Qatar; 5 Epidemiology and Biostatistics Administration, King Fahad Specialist Hospital, Dammam, Saudi Arabia; 6 Department of Neuroradiology, Hamad Medical Corporation, Doha, Qatar; 7 Clinical Radiology, Medication Education, Weill Cornell Medicine Qatar, Doha, Qatar; 8 Division of Neuroradiology, Rush University Medical Center, Chicago, IL, USA

**Keywords:** multiple sclerosis, epilepsy, corneal confocal microscopy, neurodegeneration, biomarker

## Abstract

**Purpose:**

To assess whether corneal nerve analysis can identify and differentiate patients with multiple sclerosis (MS) from those with epilepsy.

**Methods:**

Participants with MS (*n* = 83), participants with epilepsy (*n* = 50), and healthy controls (HCs) (*n* = 20) underwent corneal confocal microscopy (CCM) and quantification of automated corneal nerve fiber length (ACNFL), automated corneal nerve fractal dimension (ACNFrD), and ACNFrD/ACNFL ratio of the subbasal nerve plexus.

**Results:**

ACNFL (MS: *P* < 0.0001; epilepsy: *P* = 0.002) and ACNFrD (MS: *P* < 0.0001; epilepsy: *P* = 0.025) were significantly lower and the ACNFrD/ACNFL ratio (MS: *P* < 0.0001; epilepsy: *P* = 0.018) was significantly higher compared to HCs. ACNFL (*P* = 0.001), ACNFrD (*P* = 0.0003), and ACNFrD/ACNFL ratio (*P* = 0.006) were significantly lower in patients with MS compared to those with epilepsy. ACNFL had the highest diagnostic utility for identifying patients with MS (sensitivity/specificity 0.86/0.85, area under the curve [AUC] 0.90, *P* < 0.0001), and ACNFrD had the highest diagnostic utility for identifying patients with epilepsy (sensitivity/specificity 0.78/0.75, AUC 0.76, *P* = 0.0008). ACNFrD had the highest diagnostic utility for differentiating patients with MS from epilepsy (sensitivity/specificity 0.66/0.65, AUC 0.70, <0.0001).

**Conclusions:**

Corneal neurodegeneration occurs in and is characterized by a distinct pattern that differentiates patients with MS and epilepsy.

**Translational Relevance:**

CCM identifies and differentiates patients with MS and epilepsy, albeit with moderate performance. Further validation, with a larger sample size, is needed.

## Introduction

Neurodegeneration is a key underlying feature of neurodegenerative diseases. In multiple sclerosis (MS), brain magnetic resonance imaging identifies inflammatory lesions but does not fully account for disease progression as neurodegeneration can occur without inflammation.^1^^,^^2^ Epilepsy is characterized by recurrent unprovoked seizures with underlying neurodegeneration and gliosis, particularly in patients with hippocampal sclerosis and cortical atrophy.^3^ Magnetic resonance imaging, positron emission tomography, and magnetoencephalography can detect abnormalities associated with epileptogenic lesions,^4^ and recent investigations show that neuropathology extends beyond the lesional region.^5^ Indeed, the ENIGMA international consortium study showed alterations in hippocampal gray matter and extrahippocampal cortical regions, including the precentral and paracentral gyri in epilepsy.^6^

Optical coherence tomography (OCT) has shown retinal neuroaxonal loss in MS^7^ and epilepsy^8^ and related it to clinical disability and seizure frequency. Corneal confocal microscopy (CCM) is a reproducible and highly sensitive measure of neurodegeneration in diabetic^9^ and other peripheral neuropathies^10^ and central neurodegenerative diseases, including MS, Parkinson's disease, and dementia.^11^^–^^15^ Notably, corneal nerve degeneration has been associated with the severity of neurological disability and a higher risk of progression to dementia with a superior diagnostic performance compared to magnetic resonance imaging.^14^^–^^17^

Fractal dimension (FD) is an index of tissue structural complexity^18^ that enables the identification of unique patterns of neurodegeneration in different neurological diseases. Indeed, FD analysis of white and gray matter morphology has identified abnormalities in normal-appearing magnetic resonance imaging scans in patients with MS.^19^ In a longitudinal study,^20^ higher FD in cerebral white matter was associated with better cognitive capacity and was a stronger predictor of cognitive change over time compared to standard magnetic resonance imaging. In patients with Parkinson's disease, higher FD was associated with disease severity,^21^ and studies have also shown the utility of assessing FD in stroke^22^ and epilepsy.^23^

We previously developed and applied automated corneal nerve fractal dimension analysis (ACNFrD) to differentiate patients with and without diabetic neuropathy^24^ and also differentiated patients with diabetic neuropathy from those with chronic inflammatory demyelinating neuropathy, HIV- and chemotherapy-associated sensory neuropathy.^25^ Thus far, the utility of ACNFrD to differentiate central neurological diseases has not been assessed. In the present study, we assessed whether ACNFrD could differentiate patients with MS from those with epilepsy.

## Methods

### Study Subjects

Patients with MS (*n* = 83), patients with epilepsy (*n* = 50), and 20 healthy age-matched controls (HCs) underwent clinical, demographic, and CCM assessment. This study adhered to the tenets of the Declaration of Helsinki and obtained approvals from the Institutional Review Boards of Weill Cornell Medicine–Qatar (WCM-Q) (#15-00064 and #19-00028) and Hamad Medical Corporation (#15218/15 and #01-18-190), respectively. All patients provided informed written consent prior to participation. Patients with other diseases known to affect the corneal nerve fibers (e.g., diabetes, neoplastic or paraneoplastic disease, rheumatological disease, autoimmune disease, stroke, dementia, dry eye disease, regular contact lens use, corneal dystrophies, ocular trauma, glaucoma, or ocular surgery) were excluded.

### Corneal Confocal Microscopy

All patients underwent CCM with a Heidelberg Retinal Tomograph III Rostock Cornea Module (Heidelberg Engineering GmbH, Heidelberg, Germany) as per our previously established methodology.^14^ To perform CCM, 0.4% benoxinate hydrochloride (Minims Eye drops; Bausch & Lomb, Kingston-Upon-Thames, UK) was used as a local anesthetic, and viscoelastic gel (carbomer 980; Bausch & Lomb) was applied on both the applanating cap (TomoCap; Heidelberg Engineering) and the participant to allow optical coupling between the objective lens and the cornea. Participants were instructed to fixate on an external light target, and multiple images of the central subbasal nerve plexus were captured for each eye. Images from both eyes were acquired using the “section” mode in the Heidelberg Eye Explorer software, and each image had a field of view of 400 × 400 µm (384 × 384 pixels). Three high-clarity images per eye were selected based on depth, anatomical position, and clarity.

### Image Analysis

**Figure 1.**
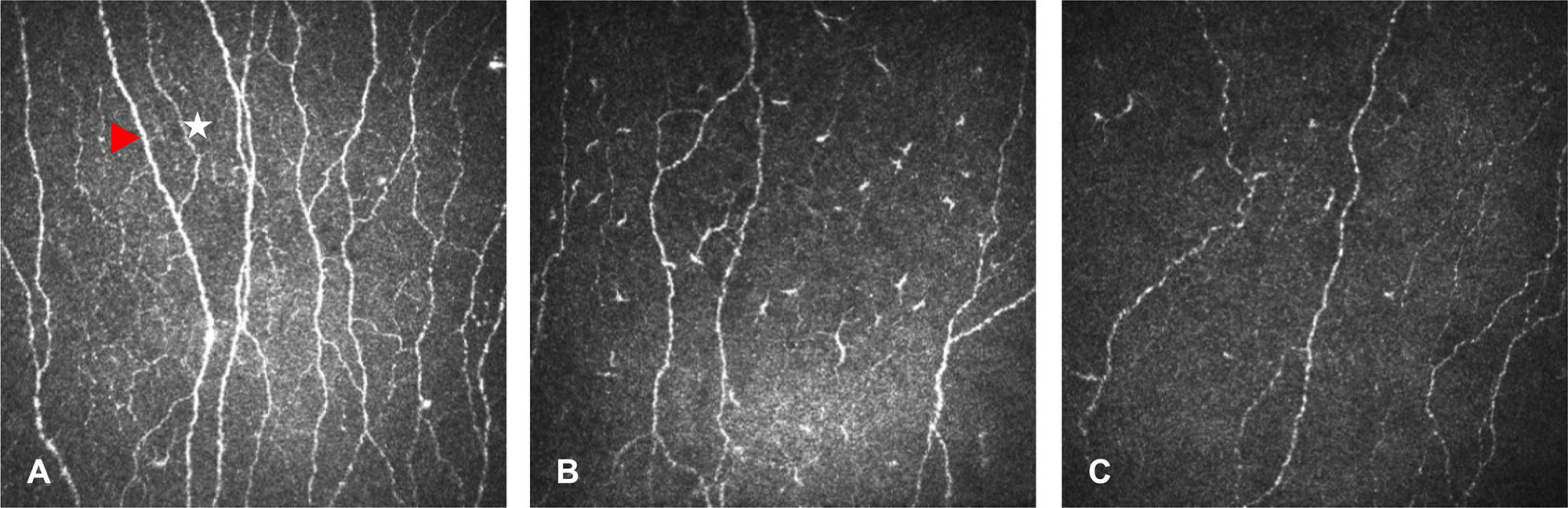
Representative CCM images. Panel shows an image from the corneal subbasal nerve plexus of an HC (**A**) with abundant main nerve fibers (*red arrowhead*) and branches (*white star*) and patients with MS (**B**) and epilepsy (**C**) with significant loss of main nerve fibers and branches. CCM images from patients with MS and epilepsy have comparable ACNFL (16.31 and 16.59 mm/mm^2^, respectively) and markedly different ACNFrD (1.43 and 1.48, respectively) values, potentially indicating a different underlying complexity pattern.

CCM images of the subbasal nerve plexus were analyzed using a validated, automated machine learning algorithm (ACCMetrics, courtesy of Professor R.A. Malik, available for free for academic users via Weill Cornell Medicine–Qatar technology licensing website: https://weillcornell.az1.qualtrics.com/jfe/form/SV_6o2ji0suM4jQinb). Three parameters were quantified: automated corneal nerve fiber length (CNFL) (mm/mm^2^), ACNFrD, and the ACNFrD to ACNFL ratio (ACNFrD/ACNFL). ACNFrD calculation was based on the box counting method,^25^ with a higher value indicating greater complexity and a lower value indicating altered morphology due to fewer, shorter, or disrupted subbasal nerves. Representative images from an HC, a patient with MS, and a patient with epilepsy are presented in Figure 1.

### Statistical Analysis

Statistical analysis was performed with SPSS (version 27 for Mac OS; IBM Corp, Armonk, NY, USA) and Prism (version 10 for Mac OS; GraphPad Software, San Diego, CA, USA). Data were confirmed to follow a normal distribution by means of a Shapiro–Wilk test (*P* > 0.05) and relevant histograms (Q-Q plots). Using a paired *t*-test and CNFL as the primary endpoint, we estimated that a sample of 80 participants with MS and 40 with epilepsy would give 90% power with a type I error of 0.05. One-way analysis of variance (post hoc Bonferroni) or nonparametric equivalent (Kruskal–Wallis test) was used for multiple comparisons between healthy controls and patients with MS and epilepsy. Receiver operating characteristic curve analysis with the area under the curve (AUC) and sensitivity and specificity identified using Youden's index was undertaken. The results are expressed as mean difference ± standard error of mean difference and *P* value unless otherwise specified. A two-sided *P* < 0.05 was considered significant.

### Data Availability

All anonymized, individual-level data used in this article are available on request to the corresponding author.

## Results

### Demographics

There was no significant difference in age and HbA1c between HCs and patients with MS and epilepsy (Table 1). There were more females in the MS group (26 men, 57 women) compared to those with epilepsy (29 men, 21 women) and HCs (9 men, 11 women). Vitamin B_12_ was significantly higher in patients with MS compared to HCs (*P* = 0.048) and those with epilepsy (*P* = 0.036), but it was within normal range. Among those with MS, 72.6% (*n* = 61) had relapsing-remitting MS and 26.2% (*n* = 22) had secondary progressive MS, with a mean disease duration of 7.02 ± 3.93 years and mean expanded disability status scale score of 1.85 ± 2.40. Among those with epilepsy, 32% (*n* = 16) had generalized epilepsy and 68% (*n* = 34) had temporal lobe epilepsy with or without mesial temporal sclerosis, with a mean disease duration of 14.61 ± 8.27 years. At the time of assessment, 4% (*n* = 2) of patients had controlled, 10% (*n* = 5) partial complex, 40% (*n* = 20) myoclonic, and 46% (*n* = 23) tonic–clonic seizures. Most patients (83%; *n* = 70) with MS were on disease-modifying treatment and 96% (*n* = 48) of patients with epilepsy were on adjunctive treatment.

**Table 1.** Demographic and Clinical Characteristics of Controls and Patients

	Study Population			*P* Value (vs. HC)		
Characteristic	HC (*n* = 20)	Epilepsy (*n* = 50)	MS (*n* = 83)	Epilepsy	MS	*P* Value (vs. MS) Epilepsy
Age (years)	36.62 ± 9.41	34.50 ± 9.30	36.91 ± 9.05	1.00	1.00	1.00
HbA1c (%)	5.62 ± 0.48	5.37 ± 0.25	5.51 ± 0.63	0.454	0.633	0.999
Vitamin B_12_ (pmol/L)	252.5 ± 101.56	257.92 ± 100.35	343.83 ± 177.80	0.999	0.048	0.036
ACNFL (mm/mm^2^)	17.96 ± 2.49	15.14 ± 3.41	13.15 ± 2.89	0.002	<0.0001	0.001
ACNFrD	1.50 ± 0.02	1.49 ± 0.03	1.47 ± 0.03	0.025	<0.0001	0.0003
ACNFrD/ACNFL	0.09 ± 0.01	0.10 ± 0.02	0.12 ± 0.03	0.018	<0.0001	0.006

Data are expressed as mean ± standard deviation.

### Corneal Confocal Microscopy

**Figure 2.**
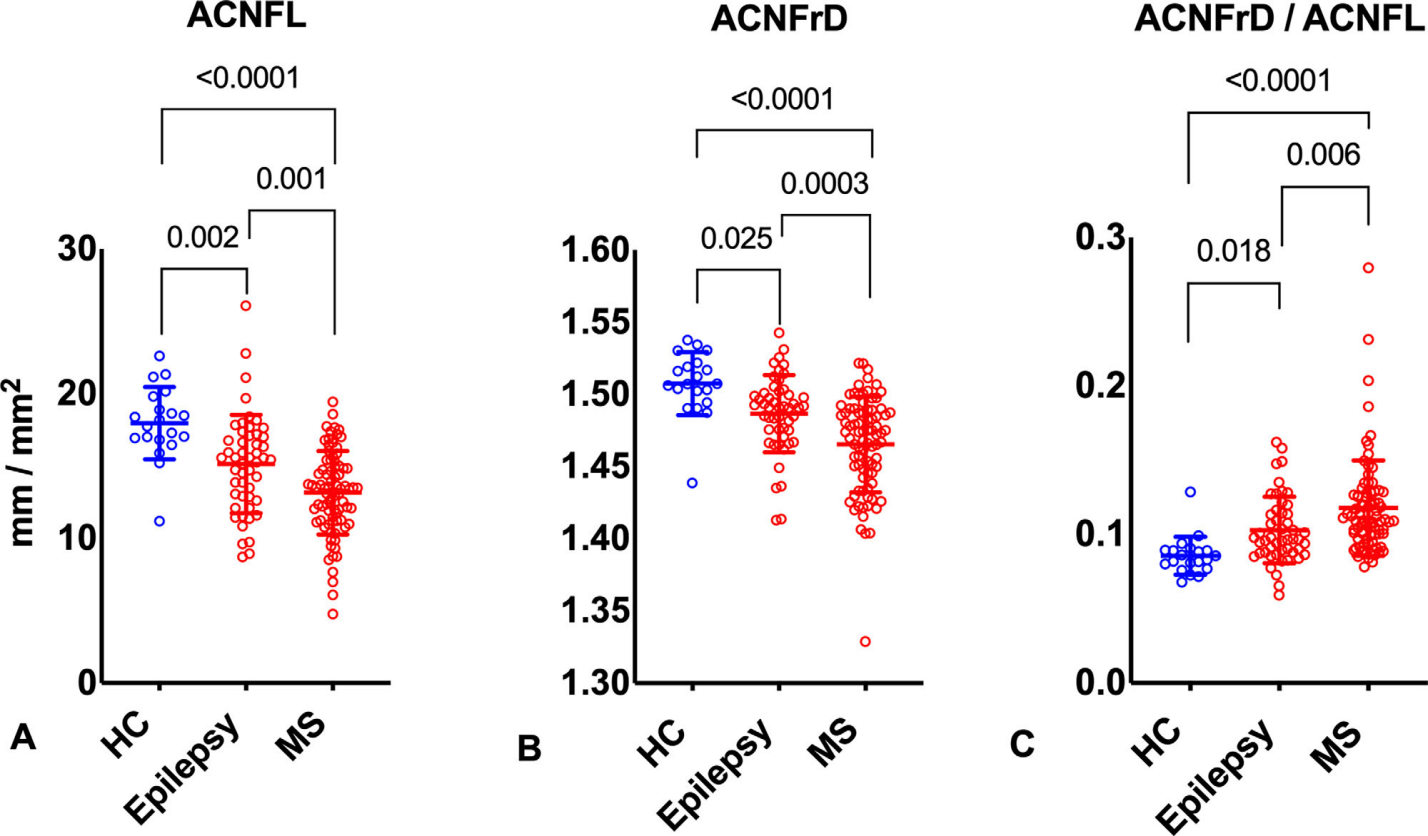
Graphic illustration of CCM data. The plots indicate full range (*dots*) with mean ± standard deviation (*overlapping lines*) for ACNFL (**A**), ACNFrD (**B**), and ACNFrD/ACNFL ratio (**C**). Data from HCs are presented in *blue*, and data from patients with epilepsy or MS are in *red*.

ACNFL (13.15 ± 2.89, *P* < 0.0001; 15.14 ± 3.41, *P* = 0.002 vs. 17.96 ± 2.49) and ACNFrD (1.47 ± 0.03, *P* < 0.0001; 1.49 ± 0.03, *P* = 0.025 vs. 1.51 ± 0.02) were significantly lower, and ACNFrD/ACNFL ratio (0.12 ± 0.03, *P* < 0.0001; 0.10 ± 0.02, *P* = 0.018 vs. 0.09 ± 0.01) was significantly higher in patients with MS and epilepsy compared to controls, respectively. ACNFL (*P* = 0.001), ACNFrD (*P* = 0.0003), and ACNFrD/ACNFL (*P* = 0.006) were significantly lower in patients with MS compared to those with epilepsy (Table 1 and Fig. 2).

### Subgroup Analysis

#### MS

In patients with relapsing-remitting MS compared to controls, ACNFL (13.53 ± 2.66 vs. 17.96 ± 2.49, *P* < 0.0001) and ACNFrD (1.47 ± 0.03 vs. 1.51 ± 0.02, *P* < 0.0001) were significantly lower, and ACNFrD/ACNFL ratio (0.11 ± 0.02 vs. 0.09 ± 0.01, *P* < 0.0007) was significantly higher. In patients with secondary progressive MS compared to controls, ACNFL (12.11 ± 3.29 vs. 17.96 ± 2.49, *P* < 0.0001) and ACNFrD (1.45 ± 0.04 vs. 1.51 ± 0.02, *P* < 0.0001) were significantly lower, and ACNFrD/ACNFL (0.13 ± 0.05 vs. 0.09 ± 0.01, *P* < 0.0007) was significantly higher. Furthermore, in patients with secondary progressive MS compared to relapsing-remitting MS, ACNFL (*P* < 0.042) and ACNFrD (*P* = 0.0392) were significantly lower, and ACNFrD/ACNFL (*P* = 0.012) was significantly higher.

#### Epilepsy

In patients with generalized epilepsy compared to controls, ACNFL (14.83 ± 3.29 vs. 17.96 ± 2.49, *P* = 0.005) was significantly lower and ACNFrD/ACNFL ratio (0.10 ± 0.02 vs. 0.09 ± 0.01, *P* = 0.004) was significantly higher with no significant difference in ACNFrD. In patients with temporal lobe epilepsy compared to controls, ACNFL (14.99 ± 3.25 vs. 17.96 ± 2.49, *P* = 0.0007) and ACNFrD (1.48 ± 0.03 vs. 1.51 ± 0.02, *P* = 0.002) were significantly lower, and ACNFrD/ACNFL ratio (0.10 ± 0.02 vs. 0.09 ± 0.01, *P* = 0.0009) was significantly higher. There was no significant difference in ACNFL, ACNFrD, or ACNFrD/ACNFL between patients with temporal lobe epilepsy and generalized epilepsy. Furthermore, ACNFL and ACNFrD were significantly lower in patients with temporal lobe epilepsy without (15.26 ± 3.60 vs. 17.96 ± 2.49, *P* = 0.013 and 1.48 ± 0.03 vs. 1.51 ± 0.02, *P* = 0.006, respectively) and with mesial temporal sclerosis (14.60 ± 2.75 vs. 17.96 ± 2.49, *P* = 0.007 and 1.48 ± 0.03 vs. 1.51 ± 0.02, *P* = 0.022, respectively) compared to controls, while ACNFrD/ACNFL was significantly higher (0.10 ± 0.02 vs. 0.09 ± 0.01, *P* = 0.006 and 0.11 ± 0.02 vs. 0.09 ± 0.01, *P* = 0.004, respectively).

### Receiver Operating Characteristics Curve Analysis

**Figure 3.**
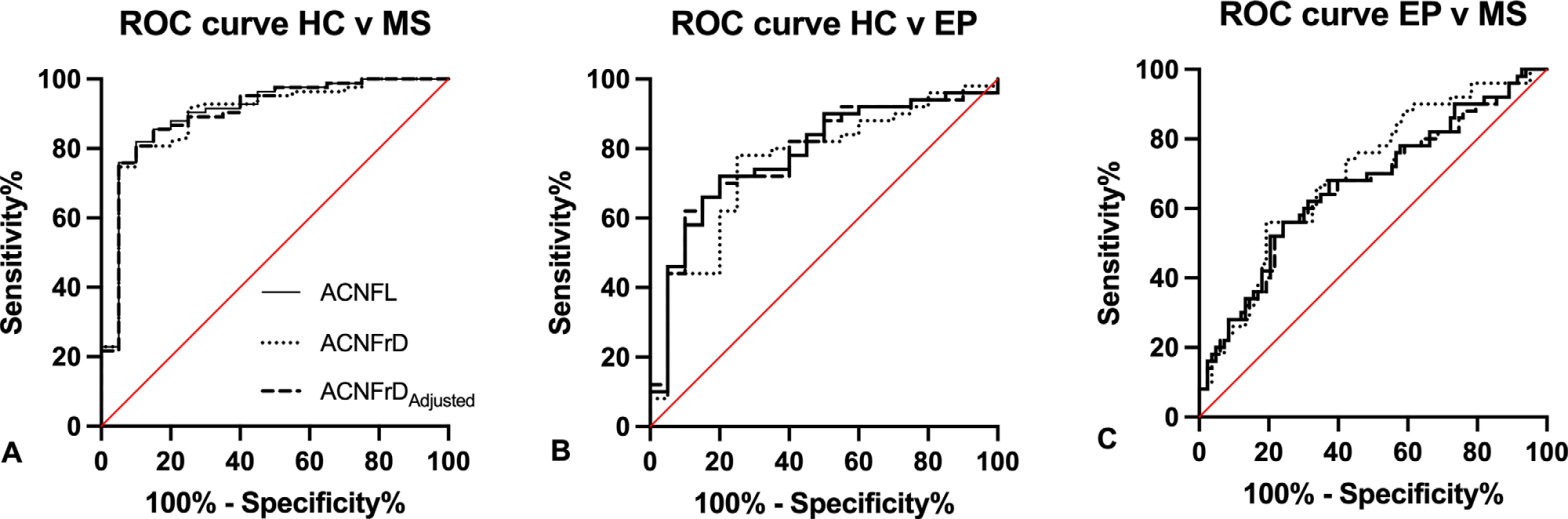
Diagnostic performance analysis. Receiver operating characteristic curves illustrating the capacity of ACNFL, ACNFrD, and ACNFrD/ACNFL ratio to distinguish between HCs and patients with MS (**A**), HCs and patients with epilepsy (**B**), and patients with epilepsy and MS (**C**).

ACNFL (sensitivity/specificity 0.86/0.85, AUC 0.90, *P* < 0.0001), ACNFrD (0.82/0.80, 0.90, *P* < 0.0001), and ACNFrD/ACNFL ratio (0.86/0.85, 0.90, *P* < 0.0001) distinguished patients with MS from HCs. ACNFL (0.74/0.70, 0.78, *P* = 0.0002), ACNFrD (0.78/0.75, 0.76, *P* = 0.0008), and ACNFrD/ACNFL ratio (0.72/0.75, 0.78, *P* = 0.0002) distinguished patients with epilepsy from HCs. Furthermore, ACNFL (0.64/0.64, 0.67, *P* = 0.001), ACNFrD (0.66/0.66, 0.70, *P* = 0.0001), and ACNFL/ACNFrD ratio (0.64/0.65, 0.66, *P* = 0.002) distinguished patients with MS from those with epilepsy (Table 2 and Fig. 3).

**Table 2.** Receiver Operating Characteristic Curve Analysis

Characteristic	Sensitivity	Specificity	LR	AUC	95% CI	*P* Value
ACNFL						
HC vs. MS	0.86	0.85	5.70	0.90	0.82–0.98	<0.0001
HC vs. epilepsy	0.74	0.70	2.47	0.78	0.67–0.9	0.0002
MS vs. epilepsy	0.64	0.64	1.77	0.67	0.57–0.77	0.001
ACNFrD						
HC vs. MS	0.82	0.80	4.09	0.90	0.81–0.98	<0.0001
HC vs. epilepsy	0.78	0.75	3.12	0.76	0.63–0.88	0.0008
MS vs. epilepsy	0.66	0.66	1.95	0.70	0.61–0.79	0.0001
ACNFrD/ACNFL						
HC vs. MS	0.86	0.85	5.70	0.90	0.82–0.98	<0.0001
HC vs. epilepsy	0.72	0.75	2.88	0.78	0.67–0.90	0.0002
MS vs. epilepsy	0.64	0.65	1.83	0.66	0.56–0.76	0.002

LR, likelihood ratio.

## Discussion

We demonstrate corneal axonal loss in patients with epilepsy and MS and further show that the pattern of corneal nerve loss, as captured by ACNFrD, differed between patients with epilepsy and MS. CCM also showed good diagnostic performance in discriminating patients with MS and epilepsy from controls and between patients with MS and those with epilepsy.

The diagnosis of epilepsy is based on the occurrence of seizures, alongside abnormalities in electroencephalography (EEG) and neuroimaging to detect epileptogenic lesions.^26^ The underlying pathology of epilepsy includes axonal loss and sprouting with altered glial function and structure.^27^^,^^28^ A recent large diffusion-weighted magnetic resonance imaging study of patients with various epilepsy syndromes showed widespread white matter abnormalities in the corpus callosum, cingulum, and external capsule^29^ and specific areas of atrophy.^30^ The fornix, which contains afferent and efferent fibers from the hippocampus, was altered in patients with epilepsy,^31^ and a histopathological study in patients with temporal lobe epilepsy showed unmyelinated axonal loss, with relative preservation of myelinated nerve fibers in the fornix and dentate gyrus of the hippocampus.^32^ Altered white matter diffusion has also been demonstrated in children with recent-onset epilepsy, implicating axonal degeneration early in the disease process.^33^ Indeed, in a recent prospective study of patients with epilepsy, spectral-domain OCT showed evidence of extensive retinal neuroaxonal loss, which was associated with the occurrence and frequency of tonic–clonic seizures and the number of antiseizure medications.^8^ Thus, in the present study, we show for the first time evidence of corneal neurodegeneration in patients with epilepsy, indicating neurodegeneration beyond the brain. We also confirm previous studies showing that CCM identifies corneal nerve degeneration in patients with MS,^34^^–^^37^ differentiates subtypes,^38^^–^^40^ and predicts disease progression.^41^^,^^42^

FD describes a shape that is self-similar across space or time,^43^ and as neurodegeneration progresses, one would predict this disrupts tissue structure and shape to affect FD. We have employed the widely used box counting method^44^ to estimate corneal nerve FD in CCM images^25^ and utilized ACNFrD as an objective measure of geometric complexity of the corneal subbasal nerve plexus in patients with diabetic neuropathy.^24^ We also previously showed that ACNFrD could differentiate patients with diabetic neuropathy from chronic inflammatory demyelinating neuropathy, HIV neuropathy, and chemotherapy-induced peripheral neuropathy.^25^ Other studies have also assessed nerve fractals in fungal keratitis,^45^ ocular surface neuropathic pain,^46^ migraine,^11^ and immune-mediated dry eye disease.^47^ In the present study, the overall severity of corneal nerve loss and the FD of the remaining nerve fibers differed significantly between patients with epilepsy and MS, even after adjusting for the severity of corneal nerve loss. Furthermore, CCM measurements showed good sensitivity and specificity for detecting and further differentiating patients with MS from those with epilepsy. The higher fractal dimension in MS compared to epilepsy, after adjusting for overall nerve loss, may reflect a different pattern of nerve damage and remodeling in MS compared to epilepsy. In the current study, we also show that the severity of corneal nerve loss and fractal dimension of the remaining fibers differs between relapsing remitting and secondary progressive MS. However, the severity of corneal nerve loss and fractal dimension of the remaining fibers did not differ between generalized and temporal lobe epilepsy with and without mesial temporal sclerosis.

Our study has strengths and limitations. The fractal pattern of corneal nerve fibers needs to be considered in relation to age and the presence of other neurological conditions. In the present study, none of the patients reported overt “dry eye” symptoms to warrant referral to ophthalmology and had therefore not undergone testing for autoantibodies or evaluation for dry eye disease based on Schirmer's test and tear breakup time. However, our study cohort was relatively young and age-matched to controls. Use of a consistent CCM imaging protocol with a fully automated image analysis method is an additional strength. The present study is cross-sectional, which limits interpretation of the utility of ACNFrD in relation to disease progression. Neuroinflammation is a common underpinning of ophthalmic^48^ and systemic disorders^49^^,^^50^ known to affect the corneal innervation. Future studies should explore whether alterations in corneal immune cell density provide additional insights into underlying mechanisms and augment the utility of differentiating MS from epilepsy. In summary, this is the first study to show significant corneal nerve degeneration in patients with epilepsy and, furthermore, shows that ACNFrD, a measurement of nerve complexity, differentiates patients with epilepsy from those with MS.
